# Molecular inhibition of RAS signalling to target ageing and age-related health

**DOI:** 10.1242/dmm.049627

**Published:** 2022-09-16

**Authors:** Mihails Laskovs, Linda Partridge, Cathy Slack

**Affiliations:** 1 School of Biosciences, College of Health and Life Sciences, Aston University, Birmingham B4 7ET, UK; 2 Institute of Healthy Ageing, Department of Genetics, Evolution and Environment, University College London, Darwin Building, Gower Street, London WC1E 6BT, UK; 3 Max Planck Institute for Biology of Ageing, Joseph-Stelzmann-Strasse 9b, 50931 Cologne, Germany

**Keywords:** MEK inhibitor, RAS pathway, Ageing

## Abstract

The RAS/MAPK pathway is a highly conserved signalling pathway with a well-established role in cancer. Mutations that hyperactivate this pathway are associated with unregulated cell proliferation. Evidence from a range of model organisms also links RAS/MAPK signalling to ageing. Genetic approaches that reduce RAS/MAPK signalling activity extend lifespan and also improve healthspan, delaying the onset and/or progression of age-related functional decline. Given its role in cancer, therapeutic interventions that target and inhibit this pathway's key components are under intense investigation. The consequent availability of small molecule inhibitors raises the possibility of repurposing these compounds to ameliorate the deleterious effects of ageing. Here, we review evidence that RAS/MAPK signalling inhibitors already in clinical use, such as trametinib, acarbose, statins, metformin and dihydromyricetin, lead to lifespan extension and to improved healthspan in a range of model systems. These findings suggest that the repurposing of small molecule inhibitors of RAS/MAPK signalling might offer opportunities to improve health during ageing, and to delay or prevent the development of age-related disease. However, challenges to this approach, including poor tolerance to treatment in older adults or development of drug resistance, first need to be resolved before successful clinical implementation.

## Abstract (precis)

**Summary:** This Review critically discusses the links between RAS signalling and ageing, and how RAS inhibitors could extend lifespan and enhance healthspan.

## Introduction

Globally, people are living longer. In developed nations, most people are expected to live well into their 70s and beyond (www.who.int/data/gho/data/themes/mortality-and-global-health-estimates/ghe-life-expectancy-and-healthy-life-expectancy). These changes are primarily due to declining mortality led by advances in hygiene, healthcare and education (Brown, 2015). However, healthspan (see Glossary, Box 1) is not keeping pace with lifespan; ageing individuals therefore experience an extended period of time of disability and ill health at the end of life. Increased age is the major risk factor for multiple chronic and debilitating diseases, including diabetes, cancer, cardiovascular disease and neurodegeneration (Niccoli and Partridge, 2012; Smetana et al., 2016; Hou et al., 2019). The prevalence of these diseases is therefore increasing worldwide alongside increased lifespans. The World Health Organization (WHO) has defined healthy ageing as the process of maintaining functional ability to enable wellbeing in older age (Rudnicka et al., 2020). The maintenance of healthy ageing therefore represents a global challenge with important socioeconomic consequences (McMaughan et al., 2020; Steptoe and Zaninotto, 2020).

Ageing has long been assumed to be an intractably complex process, caused by the accumulation of damage and pathologies because of the failure to maintain cellular integrity (López-Otín et al., 2013). However, genetic studies in model organisms have identified multiple evolutionarily conserved pathways that can be manipulated to increase lifespan and to improve multiple parameters of health during ageing. The most robust of these evolutionarily conserved longevity-associated mechanisms involve inhibition of nutrient signalling networks. For instance, genetic downregulation of insulin/IGF signalling (IIS) can increase lifespan across evolutionarily distinct organisms, including mammals (Kim and Lee et al., 2019). Moreover, genome-wide association studies in long-lived human populations have implicated a key downstream molecular effector of IIS, the FOXO3 transcription factor (Willcox et al., 2008; Deelen et al., 2019; Ji et al., 2022) (Box 2).

**Fig. 1.**
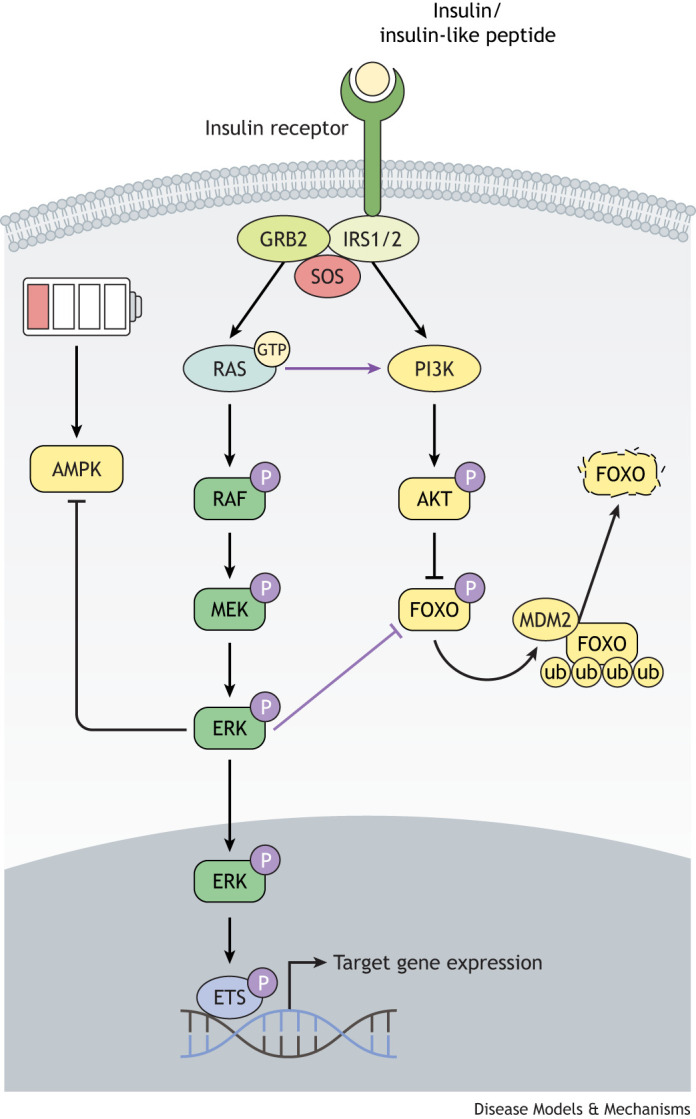
**Integration of RAS/MAPK signalling with the insulin/IGF signalling pathway.** The RAS/MAPK signalling pathway is intricately connected to the insulin/IGF signalling pathway. Upon ligand binding, the activated insulin receptor recruits IRS proteins to the intracellular receptor tail. These then assemble other adaptor proteins, including GRB2, to the activated insulin receptor complex. GRB2 then binds to and localises the RAS-GEF, SOS and, by association, activated RAS. Active GTP-bound RAS then binds to and activates its effector molecules, including RAF, initiating a phosphorylation cascade via MEK and ERK. As a key downstream effector of RAS/MAPK signalling, activated ERK phosphorylates multiple cytoplasmic and cytoskeletal proteins, including the energy-sensing AMPK. ERK can also translocate to the nucleus, where it phosphorylates and activates several transcription factors, including members of the ETS transcription factor family. In parallel, activation of PI3K leads to the phosphorylation and activation of AKT, which can translocate to the nucleus to phosphorylate FOXO. This phosphorylation results in the nuclear exclusion of FOXO, thereby preventing FOXO target gene expression. Cross-regulation of the two pathways occurs at multiple nodes: activated RAS can bind to and activate PI3K and activated ERK phosphorylates FOXO, leading to FOXO degradation via the ubiquitin proteosome system. AMPK, adenosine monophosphate-activated protein kinase; ERK, extracellular signal-regulated kinase; ETS, E-twenty-six; FOXO, Forkhead box class O; GEF, guanine nucleotide exchange factor; GRB2, growth factor receptor-bound protein 2; GTP, guanosine triphosphate; IGF, insulin-like growth factor; IRS, insulin receptor substrate; MAPK, mitogen-activated protein kinase; MDM2, mouse double minute 2 homologue; MEK, mitogen-activated protein kinase kinase; P, phosphorylation; PI3K, phosphoinositide-3 kinase; SOS, son of sevenless; ub, ubiquitin.

At the molecular level, there is extensive crosstalk between IIS and the RAS/MAPK signalling pathway, resulting in a complex nutrient signalling network (Fig. 1). RAS small GTPases are activated in response to activation of receptor tyrosine kinases (RTKs; Box 1), including the insulin receptor (IR). As GTPases, RAS proteins function as binary molecular switches, cycling between inactive GDP-bound and active GTP-bound states, coordinated by the competing activities of guanine nucleotide exchange factors (GEFs) and GTPase activating proteins (GAPs) (Rajalingam et al., 2007). Activated RTKs recruit growth factor receptor-bound protein 2 (GRB2) to the intracellular surface of the cell membrane either directly or via association with the Src homology and collagen (SHC) adaptor protein. GRB2 then binds to and localises the RAS-GEF, son of sevenless (SOS) and, by association, activated RAS to the activated RTK-bound complex. Active GTP-bound RAS then binds to and activates its effector molecules, including RAF, initiating a phosphorylation cascade via MEK (also known as MAP2K) and the extracellular signal-regulated kinase (ERK) [also known as mitogen-activated protein kinase (MAPK)]. As a key downstream effector of RAS/MAPK signalling, activated ERK phosphorylates multiple cytoplasmic and cytoskeletal proteins and can also translocate to the nucleus, where it phosphorylates and activates several transcription factors, including members of the E-twenty-six (ETS) transcription factor family (Wasylyk et al., 1998).

**Fig. 2.**
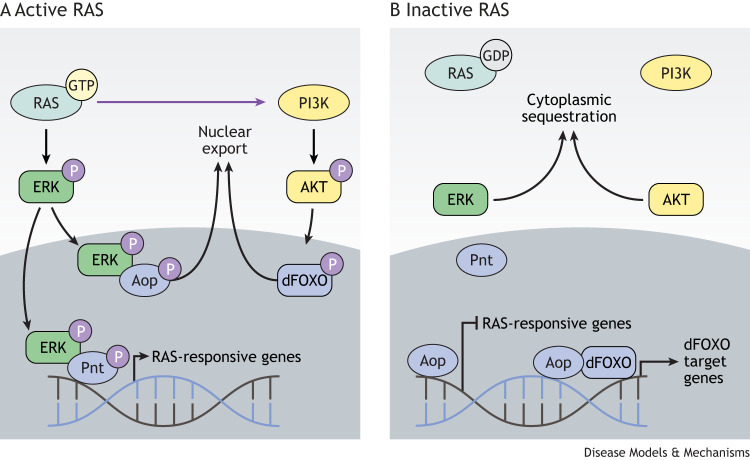
**Regulation of the ETS transcription factors Aop and Pnt in response to RAS/MAPK signalling in *Drosophila*.** (A) Activation of RAS/MAPK signalling leads to the phosphorylation and activation of ERK, which translocates to the nucleus and interacts with the ETS transcription factors Aop and Pnt. Phosphorylation by ERK activates Pnt, which then induces the expression of RAS-responsive genes. Conversely, phosphorylation of Aop inactivates it, preventing its association with the regulatory regions of its target genes and promotes its nuclear exclusion. In parallel, activation of PI3K/AKT signalling leads to phosphorylation of dFOXO and promotes its nuclear exclusion. (B) When RAS/MAPK signalling is inactive, ERK is not phosphorylated and therefore does not enter the nucleus, which leads to inactivation of Pnt and activation of Aop. Aop functions as a transcriptional repressor of RAS-responsive genes. In parallel, inactivation of PI3K/AKT leads to loss of dFOXO phosphorylation. In the absence of phosphorylation, dFOXO remains in the nucleus, where is cooperatively binds with Aop to mediate the expression of dFOXO target genes. Aop, Anterior open; dFOXO, *Drosophila* Forkhead box class O; ERK, extracellular signal-regulated kinase; ETS, E-twenty-six; GDP, guanosine diphosphate; GTP, guanosine triphosphate; MAPK, mitogen-activated protein kinase; P, phosphorylation; PI3K, phosphoinositide-3 kinase; Pnt, Pointed.

The ETS transcription factors are conserved across animals. They all contain an ∼85 amino acid ETS domain that folds into a winged helix-turn-helix DNA-binding motif that binds DNA at 5′-GGA(A/T)-3′ ETS-binding motifs (EBMs) (Dobson et al., 2019). ETS transcription factors generally function as activators, but a few repress transcription, and different family members can be differentially regulated by activated ERK. For example, the ETS transcription factors Pointed (Pnt) and Anterior open (Aop) are key transcriptional effectors of RAS/MAPK signalling during *Drosophila* development (Boisclair Lachance et al., 2014). They regulate the expression of the same set of genes, but with opposing outcomes (Dobson et al., 2019). In *Drosophila*, Pnt is activated in response to RAS signalling and functions as a transcriptional activator, whereas Aop is activated by RAS inhibition and represses gene expression (Fig. 2).

Cross-regulation between RAS/MAPK signalling and IIS occurs at multiple nodes downstream of the activated IR. For example, activated RAS-GTP can bind directly to and allosterically activate the catalytic subunit of phosphoinositide-3 kinase (PI3K) (Castellano and Downward, 2011), and, during cell growth and proliferation in *Drosophila*, this interaction allows for maximal PI3K activation (Orme et al., 2006). Conversely, AKT can negatively regulate ERK activation by phosphorylating inhibitory sites in RAF, sequestering it within the cytosol away from RAS and MEK (Zimmerman and Moelling, 1999). Moreover, activated ERK directly binds to and phosphorylates FOXO transcription factors (Box 2) on conserved serine residues, targeting them for degradation via an MDM2-mediated ubiquitin-proteasome pathway (Yang et al., 2008).

Increasing evidence from across different model organisms has shown that, like the IIS pathway, inhibition of RAS/MAPK signalling can modulate both ageing and a range of age-associated phenotypes (Slack, 2017). Hyperactivating mutations within key RAS/MAPK signalling components are prevalent in human cancers, and this has led to the development of small molecule inhibitors of the pathway for use as cancer therapeutics (Yaeger and Corcoran, 2019; Degirmenci et al., 2020). The link between reduced RAS/MAPK signalling and longevity assurance mechanisms has raised the question as to whether these compounds could be repurposed as geroprotectors (Box 1) to improve multiple parameters of health during ageing. This link also highlights the possibility of using other compounds that do not interact directly with RAS/MAPK pathway components but nevertheless indirectly inhibit the pathway's activity as geroprotectors.

Here, we review recent genetic evidence from model organisms that supports a role for RAS/MAPK signalling in ageing and discuss the impact of chemically inhibiting RAS/MAPK signalling, both directly and indirectly, on age-related health. We discuss the potential risks raised by this approach to improve healthy ageing and address key challenges for further clinical studies.

**Box 1. Glossary**

**Allosteric inhibitor:** molecule that binds to an enzyme at a site other than the active site, often resulting in a conformational change within the enzymes structure to decrease its activity.

**Autophagy:** process of conserved degradation of the cell that removes unnecessary or dysfunctional components through lysosome-dependent mechanisms.

**Fat body:** tissue in *Drosophila* with analogous function to the liver and white adipose tissue of mammals, serving as a key dynamic tissue to control energy storage and utilisation to meet energy demands of the fly.

**Geranylgeranylation:** form of prenylation involving the transfer of geranylgeranyl moiety to cysteine residues within the C-terminus of specific proteins to act as a membrane anchor for proteins.

**Geroprotectors:** drugs or compounds that target fundamental mechanisms of ageing and could delay the onset of age-related disease.

**Healthspan:** the number of years that an organism lives or can expect to live in reasonably good health.

**Prenylation**: post-translational modification of proteins involving the transfer of farnesyl or geranylgeranyl moiety to C-terminus of the target protein. Important process to mediate protein–membrane interactions.

**Proteostasis:** homeostatic maintenance of the proteome by regulating protein synthesis, translocation, post-translational modifications, protein folding and degradation.

**Receptor tyrosine kinases (RTKs):** a class of cell-surface receptors that bind to ligands such as growth factors, cytokines and hormones to initiate intracellular signalling cascades by phosphorylation of molecular effectors.

**Repurposing:** a strategy for identifying new uses for approved or investigational drugs that are outside the scope of the original medical indication.

**Box 2. FOXO transcription factors as key effectors of pro-longevity signalling**

The Forkhead box O (FOXO) family of transcription factors are key transcriptional effectors of insulin/insulin-like growth factor (IGF) signalling (IIS). This highly conserved group of transcriptional regulators comprise DAF-16 in *C. elegans*, dFOXO in *Drosophila* and four mammalian orthologues, FOXO1, FOXO3, FOXO4 and FOXO6 (Du and Zheng, 2021). They are characterised by the presence of the forkhead box, a winged-helix DNA-binding domain that binds to a consensus 5′-(A/C)AA(C/T)A-3′ recognition motif found within two sequences, the insulin-responsive element (IRE) and DAF-16 family binding element (Dai et al., 2021). Alongside the forkhead DNA-binding domain, FOXO proteins also carry a nuclear localisation signal, a nuclear export signal and a transactivation domain (Obsil and Obsilova, 2008).

IIS represses FOXO activity. Upon feeding, insulin or insulin-like peptides are released into the systemic circulation, from where they bind to the insulin receptor tyrosine kinase on the surface of target cells (Menting et al., 2013). Upon ligand binding, the insulin receptor undergoes autophosphorylation at conserved tyrosine residues within its C-terminal cytoplasmic regions. This allows for binding of insulin receptor substrates (IRS) that are subsequently phosphorylated by the tyrosine kinase activity of the activated receptor (Boucher et al., 2014). Phosphorylated IRS then act as adaptors for binding of phosphoinositide 3-kinase (PI3K), bringing PI3K in proximity to the activated receptor where it is phosphorylated and activated. PI3K then phosphorylates phosphatidylinositol (4,5)-bisphosphate (PIP2) to form phosphatidylinositol (1,4,5)-triphosphate (PIP3) at the inner surface of the plasma membrane (Boucher et al., 2014). PIP3 then binds to both 3-phosphoinositide-dependent protein kinase (PDK) and AKT (also known as protein kinase B or PKB), resulting in AKT phosphorylation, activation and release from the membrane-bound complex (Sugiyama et al., 2019). AKT is then able to translocate to the nucleus, where it phosphorylates FOXO at specific serine residues (Das et al., 2016). Phosphorylated FOXO is excluded from the nucleus through interactions with the 14-3-3 protein. Once removed from the nucleus, cytoplasmic FOXO is targeted for ubiquitination and proteasomal degradation (Huang and Tindall, 2011). Cytoplasmic FOXO can also be dephosphorylated by protein phosphatase 2A (PP2A), allowing its re-entry into the nucleus (Yan et al., 2012). Thus, AKT-dependent phosphorylation and subcellular relocation of FOXOs are key regulatory mechanisms of FOXO-dependent gene expression.

In addition to phosphorylation by AKT, FOXOs are also phosphorylated by other cellular kinases. For example, cellular stress induces activation of the c-Jun N-terminal kinase (JNK), which phosphorylates cytoplasmic FOXO at stimulatory residues, releasing it from 14-3-3 and allowing its nuclear localisation (Essers et al., 2004). AMP-activated protein kinase (AMPK) phosphorylates and activates FOXOs independently of their subcellular localisation (Greer et al., 2009), whereas activated ERK directly binds to and phosphorylates FOXOs at serine residues that target them for ubiquitin-proteasomal degradation (Yang et al., 2008). Alongside phosphorylation and ubiquitination, FOXOs are also further post-translationally regulated by acetylation, glycosylation and methylation (Wang et al., 2016). These post-translational modifications putatively serve as a context-dependent combinatorial ‘FOXO code’ to precisely regulate their activity in response to diverse external stimuli (Calnan and Brunet, 2008, Eijkelenboom and Burgering, 2013).

Activation of FOXO in response to reduced IIS is now widely accepted as the key mechanism for how IIS perturbations extend lifespan. DAF-16 and dFOXO are required for the longevity effects of reduced IIS in worms and flies, respectively (Kenyon et al., 1993, Lin et al., 2001, Slack et al., 2011). Moreover, overexpression of DAF-16 in worms or dFOXO in flies is sufficient to extend lifespan (Giannakou et al., 2004, Hwangbo et al., 2004). Once activated, FOXOs function mostly as transcriptional activators, although increasing evidence suggests that they can also repress target gene expression through interactions with protein partners (van der Vos and Coffer, 2008). Direct targets of FOXO transcriptional regulation have now been identified in worms, flies, mouse tissues and human cells by genome-wide chromatin immunoprecipitation followed by sequencing (Webb et al., 2016). These studies have revealed conserved transcriptional targets and implicate FOXO in the regulation of genes related to inflammation, metabolism, DNA repair and oxidative stress, which could all account for the evolutionary conserved role of FOXO in animal ageing. Moreover, a subset of FOXO target genes also show age-dependent changes in expression (Webb et al., 2016).

Increasing evidence implicates cooperative DNA binding by FOXO with a neighbouring transcription factor as a key mechanism that specifies target gene binding during ageing. Several of these partners have now been identified, including the ETS transcription factor Anterior open (Aop) in *Drosophila* (Alic et al., 2014). Both dFOXO and Aop are recruited to the same loci *in vivo* and both are required for IIS inhibition-mediated longevity (Dobson et al., 2019). Indeed, in the absence of Aop, activating dFOXO becomes deleterious for lifespan, suggesting that the coordinated activity of both transcription factors is required to orchestrate the appropriate response upon IIS downregulation (Dobson et al., 2019). Thus, FOXOs appear to be central to the coordination of complex cell signalling events in the longevity response.

## Single-gene mutations that extend lifespan

The first single-gene mutations reported to influence animal ageing were identified in the nematode worm *Caenorhabditis elegans*. Mutations in *age-1*, which encodes the *C. elegans* orthologue of PI3K, result in reduced function of this key effector of IIS and in a significant increase in lifespan (Friedman and Johnson et al., 1988). Subsequent mutations that decreased the activity of other core IIS molecules, including DAF-2, the worm orthologue of the insulin/IGF-1 receptor, also produced substantial increases in lifespan (Kenyon et al., 1993). The longevity effects of reducing IIS are conserved over large evolutionary distances, with similar effects on lifespan being well reported in the fruit fly, *Drosophila melanogaster*, and in mice (Tabibzadeh, 2021). Moreover, genetic variants of *FOXO3*, which encodes a key downstream effector of IIS, are strongly associated with longevity in human population studies, particularly in centenarians and in individuals over 95 years of age (Deelen et al., 2019). However, the mechanisms that underlie longevity in humans have yet to be identified.

Lifespan extension via the genetic inhibition of IIS in model organisms is often accompanied by improvements in physiological parameters of age-related health. In worms, age-related death has a multitude of causes: early death is associated with a swollen, infected pharynx and death later in life with pharyngeal atrophy. Loss-of-function *daf-2* mutations decrease death caused by pharyngeal defects (Zhao et al., 2021) and increase the mutant worms' resistance to colonisation by dietary bacteria (Podshivalova et al., 2017). Cardiac-specific overexpression of dFOXO in *Drosophila* protects against loss of cardiac function during ageing (Wessells et al., 2009). Genetic deletion of three of the *Drosophila* insulin-like peptides (dIlps) increases proteostasis (Box 1) in the gut, which maintains gut health and integrity in older flies (Tain et al., 2017). In mice, disruption of IIS activity by inhibition of growth hormone (GH; also known as GH1) release, or of GH action, reduces insulin levels, enhances insulin sensitivity, extends lifespan (Brown-Borg and Bartke, 2012), and promotes a shift in the balance between pro-inflammatory and anti-inflammatory cytokines, potentially enhancing insulin sensitivity and contributing to longevity (Salminen et al., 2011). Genetic knockout of IR substrate 1 (IRS1) in mice not only extends lifespan but is also associated with improvements in multiple markers of age-related physiological function, including motor performance, immune senescence and bone density (Selman et al., 2008).

Reducing the activity of IIS also protects against pathologies in mammalian models of age-related disease. Reduced neuronal expression of IRS2 in mice improves survival and delays the progression of motor deficits in a mouse model of Huntington’s disease (Sadagurski et al., 2011). Genetic deletion of IRS2 also suppresses amyloid deposition within the cerebral cortex in an amyloid precursor protein transgenic mouse model of Alzheimer's disease (Wakabayashi et al., 2019). Disruption of IR function using a blocking peptide or by direct transcriptional knockdown using short hairpin RNA (shRNA) inhibits the growth of cultured endocrine-resistant breast cancer cells (Chan et al., 2016a). Pharmacological interventions that diminish IIS could therefore offer wide-ranging treatments for multiple age-related pathologies.

The oncogenic RAS small GTPase is a well-known mediator of IIS in mammals that is activated when the insulin/IGF-1 RTK is stimulated (Saltiel, 2021). RAS activation induces an intracellular signal transduction cascade via the phosphorylation and activation of ERK. Furthermore, regulatory feedback from RAS and ERK modulates the activity of several key molecules within the IIS pathway, including PI3K (Castellano and Downward, 2011; Singh and Smith, 2020). Targeting the activity of RAS itself or of components of its signal transduction cascade might therefore provide novel avenues for pharmacological inhibition of a highly conserved pro-longevity pathway. Indeed, there is now increasing evidence that genetic or pharmacological modulation of signalling through this pathway shows evolutionarily conserved effects on ageing across different species.

## RAS/MAPK signalling in ageing: genetic evidence from model organisms

### Yeast

**Fig. 3.**
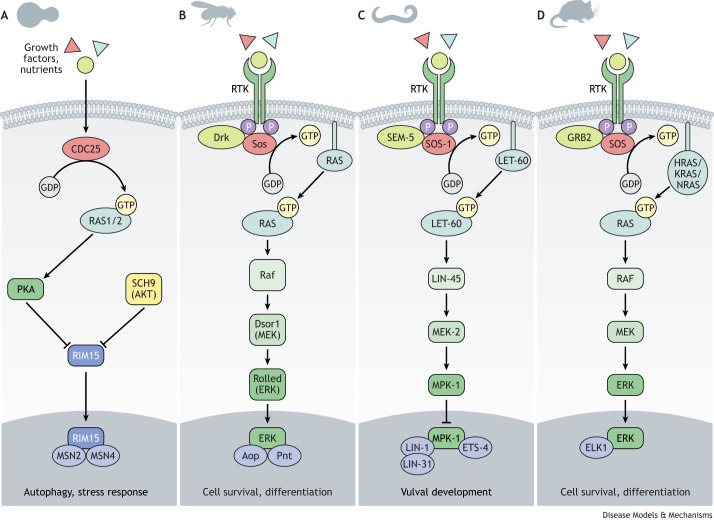
**Evolutionary conservation of the RAS/MAPK signalling pathway from yeast to mammals.** The RAS/MAPK signalling pathway, which responds to extracellular cues such as growth factors and nutrients to control cell survival, proliferation and metabolism, is highly conserved from yeast to mammals. (A-D) In this summary schematic, showing yeast (A), *Drosophila* (B), *C. elegans* (C) and mouse (D), orthologous downstream signalling components are positioned in comparable locations within the pathway and colour coded. In multicellular organisms, signalling initiates with the binding of growth factors and/or nutrients to an RTK. Across species, association of the GEF SOS with RAS promotes GDP to GTP exchange on the RAS small GTPase, activating the signalling cascade. The key downstream effector of signalling is ERK, which enters the nucleus upon activation to regulate the activity of RAS-responsive transcription factors, including members of the ETS family. Aop, Anterior open; ELK1, ETS domain-containing protein ELK-1; Drk, Dreadlocks; Dsor1, Downstream of Raf; ERK, extracellular signal-regulated kinase; ETS, E-twenty-six; GEF, guanine nucleotide exchange factor; GDP, guanosine diphosphate; GRB2, growth factor receptor-bound protein 2; GTP, guanosine triphosphate; MAPK, mitogen-activated protein kinase; MEK, mitogen-activated protein kinase kinase; P, phosphorylation; PKA, protein kinase A; Pnt, Pointed; RAF, rapidly accelerated fibrosarcoma; RTK, receptor tyrosine kinase; SOS, son of sevenless.

Ageing in the budding yeast *Saccharomyces cerevisiae* is usually assessed by the measurement of replicative and/or chronological lifespan. Replicative lifespan corresponds to the number of daughter cells produced by an individual mother cell, whereas chronological lifespan measures the survival time of non-dividing cells in the stationary phase (Mirisola et al., 2014). *S. cerevisiae* possess two RAS homologues, *RAS1* and *RAS2* (Fig. 3A). Deletion of *RAS1* extends replicative lifespan while that of *RAS2* extends chronological lifespan (Longo and Fabrizio, 2012). Similarly, genetic deletion of *CDC25*, encoding the yeast orthologue of mammalian SOS, which stimulates the nucleotide exchange of RAS proteins for their activation, results in ∼50% increase in chronological lifespan (Lin et al., 2000).

RAS signalling in yeast is responsible for relaying nutrient inputs into cell growth and division (Fontana et al., 2010). In the presence of nutrients, activation of RAS–cAMP–PKA signalling mediates the activation of a pro-growth program, which partially depends on the inhibition of the stress-responsive transcription factors MSN2/4 (Conrad et al., 2014). Conversely, downregulation of the RAS–cAMP–PKA pathway activates MSN2/4 to enhance cellular protection by activating stress responsive genes such as heat-shock proteins, superoxide dismutase, catalase and autophagy (Box 1)-related factors (Martínez-Pastor et al., 1996; Fabrizio et al., 2004; Mizuno and Irie, 2021). Activation of MSN2/4 is required for the effects of reduced RAS–cAMP–PKA signalling on chronological lifespan (Fabrizio et al., 2004) and may also be required for replicative lifespan extension in response to RAS–cAMP–PKA inhibition (Medvedik et al., 2007).

The transcriptional activity of MSN2/4 is regulated through direct phosphorylation by the serine/threonine kinase effector of PKA, RIM15 (Lee et al., 2013). RIM15 is activated in response to low RAS–cAMP–PKA signalling and is required for the chronological lifespan extension of *RAS2* deletion mutants (Wei et al., 2008). RIM15 activity is also regulated by SCH9, the yeast homologue of AKT/S6K. RIM15 translocation to the nucleus is required for RIM15-dependent activation of MSN2/4 and SCH9 directly inhibits the nuclear localisation of RIM15 through an unidentified mechanism (Pedruzzi et al., 2003). Moreover, genetic deletion of SCH9 extends replicative lifespan in yeast, which requires both RIM15 and MSN2/4 (Kaeberlein et al., 2005). RAS proteins in yeast also directly activate the adenylate cyclase CYR1, genetic deletion of which is associated with extension of chronological lifespan (Fabrizio et al., 2001).

### Drosophila

In *Drosophila*, RAS signalling functions downstream of reduced IIS in modulating lifespan. IRS proteins couple insulin/IGF-1 receptor stimulation to the activation of downstream molecules (Fig. 3B) and recruit Sos to the activated receptor through associations with the GRB2 adaptor protein [Dreadlocks (Drk)]. *Drosophila* express a single IRS protein, Chico. Mutation of Chico within the proposed Drk binding site disrupts Chico–Drk interactions *in vitro* (Slack et al., 2015). The same mutation within a transgenic genomic rescue construct failed to rescue the lifespan extension associated with *chico* null mutation (Slack et al., 2015).

Mimicking the effects of reduced RAS signalling by manipulating the expression of its transcriptional effectors, either by expression of an activated form of Aop or by RNA interference (RNAi)-mediated knockdown of Pnt within the adult *Drosophila* fat body (Box 1), is sufficient to extend lifespan (Alic et al., 2014). Aop activation is also required for the extension of lifespan induced by loss-of-function mutations in *chico* (Slack et al., 2015). Moreover, direct inhibition of RAS activity itself within the adult fly, either by RNAi or by overexpressing a dominant-negative isoform, extends lifespan in *Drosophila* (Slack et al., 2015). Inhibition of RAS signalling also alleviates the pathophysiological effects of neuronal mitochondrial dysfunction in a *Drosophila* model of familial Parkinson's disease, in which loss-of-function mutations in *Ras85D* rescued the locomotory defects of *park^25^* homozygous mutant flies (Duncan et al., 2018).

Surprisingly, the effects of RAS/MAPK suppression on neuronal mitochondrial dysfunction phenotypes were recapitulated with RNAi-mediated knockdown of either Aop or Pnt, which are generally considered act antagonistically in the canonical model of RAS/MAPK signalling. However, in *Drosophila* motor neurons, Aop and Pnt appear to both act as positive effectors (Duncan et al., 2018). Thus, although direct inhibition of RAS/MAPK signalling offers beneficial outcomes in models of neuronal mitochondrial dysfunction and during normal ageing, Aop may have different functions in these two scenarios. Indeed, RNAi-mediated knockdown of Aop specifically in adult neurons can be beneficial in models of neuronal mitochondrial dysfunction but significantly shortens lifespan in normal flies (Dobson et al., 2019). This could reflect differential regulation of Aop across different tissues. For example, Aop stability in neurons is differentially regulated by RAS/MAPK signalling depending on their differentiation state (Peláez et al., 2015). Of course, RAS/MAPK signalling has additional upstream inputs from other RTKs as well as IR activation, and these may contribute to the differential regulation of RAS/MAPK transcriptional effectors such as Aop. For example, expression of a dominant-negative isoform of Egfr, but not of IR, in the intestinal enterocytes of adult flies extends lifespan, similarly to the expression of activated Aop in the same cells (Dobson et al., 2019). Furthermore, signalling via RAS/MAPK has additional transcriptional outputs, and tissue-restricted expression or regulation of these other RAS-responsive transcription factors could contribute to lifespan extension upon reduced RAS/MAPK activity. Nevertheless, genetic inhibition of RAS/MAPK signalling in different tissues by targeting specific upstream components of the pathway, including RAS itself, is sufficient to extend lifespan, regardless of the downstream mediator (Slack et al., 2015; Duncan et al., 2018).

### C. elegans

The canonical RAS signalling pathway is also conserved in *C. elegans* (Fig. 3C) (Sternberg and Han, 1998). In worms, the RAS GTPase, encoded by the *let-60* gene, plays a role in vulval development and excretory systems (Nanji et al., 2005). Unlike in yeast and flies, reduction-of-function mutations in *let-60* alone did not extend lifespan and actually partially suppressed ­*daf-2-*dependent lifespan extension. Conversely, gain-of-function mutations in *let-60* enhanced the longevity effects of *daf-2* loss of function, increasing maximal lifespan in *daf-2* mutant worms even further (Nanji et al., 2005). An important distinction between the RAS pathway in *C. elegans* and that in other animal models is the absence of a worm Aop orthologue. Instead, signalling downstream of LET-60 may be potentiated via the ETS transcription factor LIN-1 (Tiensuu et al., 2005), which has also been implicated in the transduction of IIS downstream of the DAF-2 receptor during ageing (Dobson et al., 2019). These differences in the transcriptional output of RAS signalling in *C. elegans* may therefore account for the differential effects of RAS inhibition on lifespan.

Despite the absence of a direct orthologue of Aop in *C. elegans*, the transcription factor ETS-4 may represent a functional homologue. Epistasis analysis using loss-of-function alleles for *ets-4* suggests that it acts in a parallel pathway to DAF-2 during ageing (Thyagarajan et al., 2010). ETS-4 activity is limiting for worm lifespan and restoring ETS-4 expression specifically within the intestine in *ets-4* mutants is sufficient to rescue lifespan defects (Thyagarajan et al., 2010). Similar to Aop and dFOXO in *Drosophila*, ETS*-*4 also shares transcriptional targets with the worm FOXO orthologue, DAF-16 (Thyagarajan et al., 2010). Therefore, despite RAS signalling having different roles during ageing in *C. elegans* and flies, ETS transcription factors in these two model systems might regulate the expression of common target genes that affect lifespan.

The importance of LET-60 signalling for vulval integrity might also affect the ability of *let-60* loss of function to increase lifespan in *C. elegans*. Loss of vulval integrity leads to decreased lifespan, whereas preventing vulval defects correlates with longevity (Leiser et al., 2016). The ETS transcription factor LIN-31 is a vulval-specific effector of LET-60 signalling in worms. LIN-31 forms a complex with LIN-1, which inhibits vulval induction. However, LIN-31 is directly phosphorylated by MPK-1, the ­worm orthologue of ERK, which disrupts the LIN-1/LIN-31 complex and thus promotes vulval cell fates (Tan et al., 1998). Ubiquitous loss of LET-60 signalling in reduction-of-function mutants might therefore decrease worm lifespan through the loss of vulval integrity via disruptions to LIN-1/LIN-31 activity. Restricting the reduction of LET-60 signalling to specific tissues may therefore bypass the detrimental effects of LET-60 reduction of function in the vulva. Similar to observations in flies, the spatial activation of different transcriptional mediators of RAS/MAPK signalling could influence its capacity to extend lifespan (Dobson et al., 2019).

### Mammals

A role for the inhibition of RAS signalling in mammalian ageing has yet to be determined. Mammals have four members of the RAS protein family, NRAS, HRAS, KRAS4A and KRAS4B, which are expressed from three genes. KRAS4A and KRAS4B are isoforms expressed from the same *Kras* gene (Fig. 3D). Knockout of the entire *Kras* locus in mice is embryonic lethal (Johnson et al., 1997). However, mouse knockouts for *Nras*, *Hras* and *Kras4A*, the latter of which still expresses a functional KRAS4B protein, are adult viable. In addition, lifespan experiments have only been reported for *Kras4A* deletion, which shows no obvious effects on survival (Umanoff et al., 1995; Ise et al., 2000; Plowman et al., 2003). Indirect inhibition of HRAS has been achieved by genetic knockout of *Rasgrf1*, which encodes the GEF RASGRF1 that stimulates the GDP to GTP exchange on and subsequent activation of HRAS, as well as members of the RAS-related protein (R-RAS) and RAC families of small GTPases (Fernández-Medarde and Santos, 2011). *Rasgrf1* homozygous knockouts show ∼20% increases in both average and maximal lifespan compared to control animals (Borrás et al., 2011). Interestingly, lifespan extension was also observed in tumour-free animals, suggesting that the increased survival of *Rasgrf1* knockouts was not simply a consequence of reduced tumour incidence (Borrás et al., 2011).

In humans, mutations in *HRAS* are associated with Costello syndrome, a rare genetic condition characterised by short stature, developmental delay and distinctive facial features (Gripp et al., 2019). Costello syndrome patients also show multiple physiological disorders associated with premature ageing, including osteoporosis and osteopenia (Gripp et al., 2019). An allelic variant of *HRAS1*, consisting of a variable number of 28 bp tandem repeats downstream of the coding region, shows reduced frequency in centenarians compared to younger controls (Bonafè et al., 2002). Such alleles of *HRAS1* are proposed to contain binding sites for NF-κB, a key stress-responsive transcription factor and inflammatory mediator (Bonafè et al., 2002). Analysis of copy-number variants associated with mortality at old age identified a deletion that encompasses *HRAS1* that was consistently associated with higher mortality at older ages across three different populations (Kuningas et. al., 2011). However, this deletion is located in a gene-rich region, and other genes located here or in close proximity might also be relevant. In the search for longevity-determining genes in humans, genome-wide association studies have so far not found an association between allelic variants in RAS genes and longer lifespan. However, variants of *HRAS1* are associated with exceptional longevity when they occur in combination with certain allelic variants of two other genes, *APOE* and *LASS1* (also known as *CERS1*) (Jazwinski et al., 2010)*.* Moreover, allelic combinations of the three genes were not only associated with longevity, but also with reduced incidences of self-reported functional deficits in study participants (Jazwinski et al., 2010). Thus, genetic interactions between *HRAS1* and other longevity-associated genes such as *APOE* may play an important role in the genetic effects on ageing in humans.

In summary, emerging evidence from genetic studies in both invertebrate and mammalian models of ageing and age-related disease, as well as genomic studies in human populations, have identified a role for RAS/MAPK signalling in ageing and age-related health. Further exploration of these functions of RAS/MAPK signalling may offer new therapeutic targets to improve healthy ageing. Furthermore, pharmacological inhibition of the pathway to improve healthy ageing will be critical for clinical translation.

### From genetics to intervention

A better understanding of the genetic pathways that underlie ageing has helped to identify molecular targets for pharmacological interventions that might both extend lifespan and improve age-related health. Small molecule RAS pathway inhibitors have been developed as anti-cancer therapeutics to target hyperactivating mutations in key RAS/MAPK signalling components that drive unregulated cell proliferation, several of which are already in clinical use (Table 1). Here, we discuss the repurposing (Box 1) of these compounds, along with other molecular inhibitors of RAS/MAPK signalling, as geroprotectors. We also highlight the challenges and limitations that this repurposing raises. We have specifically chosen compounds that have demonstrated clear inhibition of RAS/MAPK signalling, either directly or indirectly, and that have also shown effects on ageing or age-related health in model systems.

**Table 1.**
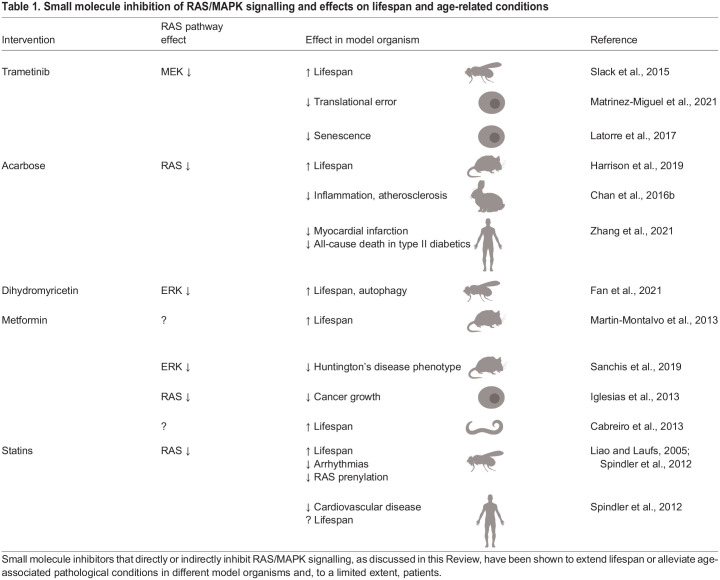
Small molecule inhibition of RAS/MAPK signalling and effects on lifespan and age-related conditions

### Trametinib

Trametinib is a specific inhibitor of MEK and is currently used clinically to treat some forms of metastatic melanomas (Hoffner and Benchich, 2018). Oral administration of trametinib to adult *Drosophila* is reported to extend their median lifespan by 8-12% when added directly to their food at concentrations of 1.56-15.6 µM (Slack et al., 2015). Moreover, feeding 15.6 µM of trametinib to flies in later life, from 30 days of age, was sufficient to extend adult flies' lifespan, albeit to a smaller extent than continual exposure. Later-life onset of treatment increased median lifespan by ∼4% (Slack et al., 2015). Trametinib is one of several clinical MEK inhibitors (Han et al., 2021), but it has yet to be determined whether its effects on *Drosophila* lifespan are drug specific. Recent structural and biochemical studies of trametinib have identified some important differences in the mechanism of action of trametinib compared to other allosteric inhibitors (Box 1) of MEK. For example, trametinib acts more potently on MEK when it is complexed with RAF rather than on free MEK (Gonzalez-Del Pino et al., 2021), whereas other MEK inhibitors induce the formation of these MEK/RAF complexes in KRAS-mutant lung cancer cell lines, which prevents durable inhibition of ERK (Lito et al., 2014). Crystal structures of trametinib bound to MEK-kinase suppressor of RAS (KSR) complexes have identified binding sites for trametinib not only in the typical allosteric binding pocket of MEK, but also where MEK interacts with KSR, an interaction that is not observed with other MEK inhibitors (Khan et al., 2020). It will therefore be interesting to determine whether the lifespan-extending effects of trametinib can be recapitulated by other MEK inhibitors.

The mechanisms by which trametinib extends lifespan in *Drosophila* are not fully understood, but recent work points to the maintenance of translational fidelity as one possibility. Loss of proteostasis is one of the widely accepted hallmarks of ageing and is affected by the accuracy of translation (López-Otín et al., 2013). In *S. cerevisiae*, treatment with paromomycin or nourseothricin, which both induce translational errors by increasing the frequencies of stop-codon read-through and amino acid misincorporation, also accelerates chronological ageing. Moreover, the same study showed that mutation of *RPS2*, encoding the 40S ribosomal protein S2, also showed increased translational errors and reduced lifespan (von der Haar et al., 2017). Mutation of the yeast *MRPS12*, which encodes the S12 orthologue of the mitoribosome, reduces the frequency of translational errors in both mitochrondrial and cytosolic proteins and extends chronological lifespan (Suhm et al., 2018). Similarly, in worms and flies, mutation of orthologues encoding RPS23, a key protein in the ribosomal decoding centre, leads to more accurate translation and longer lifespan (Martinez-Miguel et al., 2021).

Martinez-Miguel and colleagues analysed stop codon read-through and amino acid misincorporations in *Drosophila* S2R^+^ cells to show that 5 nM and 10 nM trametinib can reduce translational errors *in vitro* (Martinez-Miguel et al., 2021). However, the authors did not test whether these same doses of trametinib had any effect on lifespan, or examine translational error rates in flies treated with trametinib at doses previously determined to be conducive to lifespan extension. Moreover, the direct consequences of fewer translational errors during ageing are not well understood, and the effects of trametinib on translational error have yet to be determined in mammals.

Pharmacological inhibition of MEK using trametinib has been shown to reduce insulin resistance in obese but genetically wild-type mice and in genetically obese (*ob/ob*) mice, which suggests that RAS inhibition might affect metabolic regulation during ageing (Banks et al., 2015). Trametinib treatment is also associated with reduced cellular senescence *in vitro*, another key hallmark of ageing (López-Otín et al., 2013). Latorre and colleagues have shown that the treatment of senescent normal human dermal fibroblasts (NHDF) cells with either 1 µM or 10 µM trametinib resulted in a robust, dose-dependent decrease in the proportion of senescent cells in culture. Furthermore, treatment with trametinib was able to negate the pro-senescent effects of the ERK agonist ceramide in these cells (Latorre et al., 2017). The expression of mRNA splicing factors shows age-dependent changes in human peripheral blood leukocytes (Harries et al., 2011), and similar gene expression changes were observed by Latorre and colleagues in senescent NHDF cells (Latorre et al., 2017). Interestingly, alongside its effects on cellular senescence, trametinib treatment in NHDF cells also restored the expression of multiple splicing factors to levels seen in early-passage cultures (Latorre et al., 2017). Investigations into the effects of trametinib on cellular senescence and translational fidelity during ageing *in vivo* would therefore be interesting to explore further.

### Dihydromyricetin

The flavonoid dihydromyricetin (DHM), extracted from the *Ampelopsis* plant, has a range of antioxidant, anti-inflammatory and anti-carcinogenic effects, and has been used in traditional Chinese medicine to treat pyretic fever, cough, pain and jaundice (Li et al., 2017). Its administration in rodent models of age-related neurodegeneration improved multiple phenotypes associated with disease. For example, in both rat and mouse models of Alzheimer's disease, DHM improved cognitive function and reduced neuronal cell loss (Liang et al., 2014; Sun et al., 2019). Similarly, in mouse models of Parkinson's disease, treatment with DHM protected against loss of dopaminergic neurons and associated behavioural deficits (Ren et al., 2016; Wu et al., 2019).

In *Drosophila*, feeding adult flies with 40 μM DHM can extend median lifespan by ∼16% and maximum lifespan by ∼6%. These effects were associated with decreased levels of phosphorylated ERK and AKT, and activation of both Aop and dFOXO (Fan et al., 2021). Furthermore, similarly to trametinib, late-life exposure to 40 μM DHM from 30 days of age was also found to increase fly lifespan, although to a lesser extent than chronic exposure to the same dose throughout adulthood (Fan et al., 2021).

Autophagy is an evolutionarily conserved cellular recycling mechanism that maintains protein and organelle quality by targeting cellular components for lysosomal degradation. In worms and flies, upregulation of autophagy by overexpression of key regulatory factors increases lifespan (Barbosa et al., 2019). Pharmacological inhibition of RAS/MAPK signalling using the covalent KRAS^G12C^ inhibitor ARS-853, the MEK inhibitors trametinib or cobimetinib, or the ERK1/2 (also known as MAPK3/1) inhibitor SCH772984 increases autophagic flux (Bryant et al., 2019; Kinsey et al., 2019). These long-lived DHM-treated flies also showed increased resistance to starvation, a reduction in age-related intestinal dysfunction, and increased expression of the autophagy-related genes *Atg1*, *Atg5* and *Atg8b*, indicating that DHM might promote longevity and improve healthspan in part by upregulating autophagy (Fan et al., 2021).

### Statins

Statins lower circulating levels of low-density lipoprotein and cholesterol and prevent dyslipidaemia (Liao and Laufs, 2005), and so are often used clinically to prevent cardiovascular disease. Statins are highly specific inhibitors of HMG-CoA reductase, which is the rate-limiting enzyme of the mevalonate pathway, the metabolic pathway that produces cholesterol and other isoprenoids, such as farnesyl pyrophosphate and geranylgeranyl pyrophosphate. Such isoprenoids are required for the post-translational farnesylation and geranylgeranylation (Box 1) of proteins, and especially of small signalling GTPases, including RAS itself (Bonetti, 2003). These post-translational modifications are crucial for RAS activation and membrane anchoring via prenylation (Box 1). Statins reduce the prenylation of both RAS and Rho in endothelial cell culture, leading to the accumulation of inactive forms of both proteins in the cytoplasm (Liao and Laufs, 2005).

Oral administration of 240 µM simvastatin to adult male flies increased their mean lifespan by ∼25%, an effect that was associated with decreased levels of active farnesylated and geranylgeranylated forms of both RAS and Rab4 (Spindler et al., 2012). Furthermore, the direct inhibition of protein prenylation by L744832, a specific farnesyl transferase inhibitor, and by GGTI-298, a specific geranylgeranyl transferase inhibitor, also increased fly lifespan to a similar extent (Spindler et al., 2012). Simvastatin not only increased lifespan but also improved healthspan in *Drosophila* by decreasing heart arrhythmias in old male flies. Thus, RAS signalling inhibition by simvastatin may increase longevity by improving heart health (Spindler et al., 2012).

Simvastatin and a closely related analogue, lovastatin, also extend the lifespan of *C. elegans* at relatively low doses of 25 µM, 50 µM and 100 µM (Jahn et al., 2020). Ageing in worms is associated with the accumulation of fluorescent pigments such as lipofuscin, formed from oxidised and cross-linked proteins, lipids and carbohydrates, and advanced glycation end-products formed from the non-enzymatic addition of sugar to free amino groups of proteins (Son et al., 2019). Treatment with lovastatin significantly reduced the accumulation of these fluorescent pigments during ageing, demonstrating that statins act on biomarkers of ageing in worms, not just on lifespan (Jahn et al., 2020). In this study, the reduction of ageing pigment accumulation and lifespan extension induced by statins depended on DAF-16 activity (Jahn et al., 2020). However, direct effects on RAS prenylation in response to these treatments have yet to be established in worms.

Statins may also improve healthspan by preventing cancer. Statins activate endoplasmic reticulum stress via inhibition of RAS prenylation, leading to enhanced immune responses against KRAS-mutant cancer cell lines and against KRAS-mutant tumours in the lung and liver of genetically engineered mouse models (Nam et al., 2021). The exposure of blood cancer cells to simvastatin has been shown to decrease phosphorylated ERK levels and to suppress RAS prenylation in human acute promyelocytic leukaemia (HL-60) cells and human histiocytic lymphoma (U937) cells in culture, subsequently inducing cell death (Fujiwara et al., 2017). Furthermore, a recent epidemiological study found an association between statin use and reduced risk of cancer incidences and cancer-related morbidities in patients with heart failure (Ren et al., 2021).

### Acarbose

Acarbose is a complex oligosaccharide that acts as an inhibitor of alpha-glucosidase and alpha-amylase, digestive enzymes that are responsible for the breakdown of complex carbohydrates in the intestine. By inhibiting these enzymes, acarbose limits the absorption of dietary carbohydrates, thereby limiting associated increases in blood glucose levels and subsequent insulin release. Owing to these properties, acarbose is routinely used in conjunction with metformin, exercise and diet to treat type 2 diabetes (Martin and Montgomery, 1996; Hsu et al., 2018).

Activated RAS supports the proliferation and migration of vascular smooth muscle cells, thereby propagating atherosclerosis (Yu et al., 2018). Treating vascular smooth muscle cells (A7r5) in culture with increasing doses of acarbose for 24 h and 48 h resulted in both dose- and time-dependent inhibition of their proliferation (Yu et al., 2018). These effects on cell proliferation were associated with decreased levels of RAS protein, as well as with a reduction in phosphorylated ERK levels, indicating that acarbose decreases atherosclerosis by targeting RAS signalling (Yu et al., 2018). Acarbose is thought to regulate RAS protein expression through microRNA (miRNA)-dependent regulation of gene expression. Acarbose upregulates the expression of miR-143, a specific miRNA that targets transcripts directly to regulate expression of RAS proteins (Wang et al., 2014; Akao et al., 2018).

Confirming *in vitro* findings (Yu et al., 2018), acarbose also inhibits RAS and the development of atherosclerosis in rabbits fed a high-cholesterol diet. Oral administration of acarbose to these rabbits at 2.5 mg/kg and 5 mg/kg resulted in a significant decrease in the levels of the inflammatory marker TNF-a, as well as a dose-dependent decrease in RAS protein levels (Chan et al., 2016b). Acarbose also increases the median lifespan of male mice by 16-17% and that of female mice by 4-5% (Harrison et al., 2019). In humans, acarbose therapy significantly reduced the incidence of myocardial infarctions and all-cause deaths in patients with type 2 diabetes, suggesting that acarbose can improve health and thus potentially extend lifespan (Zhang et al., 2021). Currently, acarbose is in Phase 2 clinical trials to study its effects during ageing in individuals aged 60-100 years (Garay, 2021).

### Metformin

Metformin is a first-line medication for the treatment of type 2 diabetes, particularly in overweight individuals, and is also frequently used to treat polycystic ovary syndrome and to prevent nephrotoxicity (Nasri and Rafieian-Kopaei, 2014). Metformin functions as an anti-hyperglycaemic drug by lowering blood glucose concentration without causing hypoglycaemia. Metformin also has anti-tumour properties in RAS-dependent cancers. Exposure of human Hec1A endometrial carcinoma cells or Panc1 pancreatic cancer cells, which both carry RAS-hyperactivating mutations, to metformin *in vitro* results in the removal of the constitutively active RAS from the plasma membrane and translocation into the cytoplasm, thereby inhibiting RAS signalling and cell proliferation (Iglesias et al., 2013; Nair et al., 2014). Metformin also downregulates specificity protein (Sp) transcription factors in Panc1 cells *in vitro* to inhibit the EGF-dependent activation of RAS (Nair et al., 2014).

Recently, metformin treatment was shown to extend lifespan in several model systems. In *C. elegans*, exposure to metformin at concentrations of 50 mM and 100 mM resulted in a 40% longer median lifespan but no increase in maximum lifespan (Onken and Driscoll, 2010; Cabreiro et al., 2013). Similarly, exposure to phenformin, a closely related but more potent bioguanide, also extended lifespan in worms when used at doses of 3 mM and 4.5 mM (Cabreiro et al., 2013). The effects of metformin on worm lifespan were dependent on AMP-activated protein kinase (AMPK) activation. AMPK is a major regulator of energy metabolism and is activated in response to changes in the cellular ATP:AMP ratio (Long, 2006). Activated ERK can directly phosphorylate AMPK on Ser485 to inhibit its activity (López-Cotarelo et al., 2015; Ovens et al., 2021), while dephosphorylation of AMPK at Thr172, an activation site, is associated with stress-induced ERK activation (Kodiha et al., 2007). Thus, the effects of metformin on lifespan in *C. elegans* may result from relieving ERK-dependent AMPK inhibition.

However, these metformin effects on lifespan appear not to be conserved in *Drosophila*. Even though metformin activates AMPK, and the genetic activation of AMPK increases lifespan in *Drosophila*, metformin-treated flies did not show extended lifespan at any dose, and exhibited perturbations in intestinal homeostasis and toxicity at high doses (Stenesen et al., 2013; Slack et al., 2012). In rodent models, metformin has been shown to extend lifespan in both male and female mice, and the effects were generally greater in female mice (Anisimov et al., 2008). Similarly, chronic treatment of female C3H/Sn mice with phenformin extended both mean and maximum lifespan by ∼20% and reduced the incidence of spontaneous mammary tumours in these animals (Anisimov et al., 2003).

However, the mouse strains used in these studies were relatively short-lived and prone to developing cancer. More recent studies using longer-lived and genetically outbred strains have provided less conclusive results, suggesting that metformin's effects on lifespan might be context dependent, with more dramatic effects observed under conditions that shortened lifespan (Martin-Montalvo et al., 2013; Strong et al., 2016). Metformin might also protect against age-related disease in animal models. In a mouse model of Huntington’s disease, 20 mg/kg oral metformin per day prevented the upregulation of phosphorylated ERK in the cortex (Sanchis et al., 2019). Thus, metformin might alleviate Huntington's disease phenotypes in mice, at least in part by preventing RAS/MAPK signal induction (Sanchis et al., 2019).

## Conclusions and future perspectives

The significant increase in life expectancy within the past century poses important socioeconomic concerns, requiring novel strategies to maintain health and well-being amongst an increasingly older global population. Ageing research in model organisms has led to the rapid identification and genetic dissection of key ageing pathways, including the RAS/MAPK signalling pathway. Genetic interventions that modulate signalling through RAS proteins and their downstream effectors have been shown to increase lifespan in these model systems and to improve multiple parameters of health, both during normal ageing and in animal models of age-related disease.

RAS/MAPK signalling therefore adds to an emerging theme that manipulating cancer-promoting pathways, either by inhibiting the function of oncogenes or by increasing the activity of tumour suppressors, can affect healthy ageing. Examples from rodent models that extend lifespan include *Myc* haploinsufficiency (Hofmann et al., 2015), extra genomic copies of the *Ink4/Arf* locus, that elevate expression of its encoded tumour suppressor proteins p16 and p14 (Matheu et al., 2009), and increased gene dosage of the tumour suppressor *Pten* (Ortega-Molina et al., 2012), or mimicking its activation through genetic inhibition of its direct target, PI3K (Foukas et al., 2013. Interestingly, although some these models had reduced cancer incidence, researchers also observed lifespan extension in cancer-free animals, suggesting that delayed ageing may not simply be a consequence of protection against cancer.

Nevertheless, the prominent role of RAS/MAPK signalling in cancer has led to the isolation of several small molecule inhibitors of the pathway, and some are already in clinical use. As discussed in this Review, recent work with model organisms suggests that these same compounds may provide beneficial effects on age-related health. Thus, repurposing these anti-cancer treatments could provide a useful strategy to develop novel interventions to promote healthy ageing. Moreover, other pro-longevity pharmacological interventions may elicit their effects on ageing and age-related health, at least in part through perturbations in RAS/MAPK signal transduction. It should be noted, though, that compounds like metformin, acarbose, DHM and statins have much broader effects, and, although they have an impact on RAS/MAPK outputs, they do not exclusively target this pathway.

However, cancer therapeutics, including small molecule inhibitors of RAS/MAPK signalling, are notoriously toxic and can elicit severe side effects when administered for cancer treatment, particularly in older patients. Older cancer patients do not tolerate anti-cancer treatments well and experience increased occurrences of adverse events and side effects, like poor appetite and anaemia (de Glas, 2020). Furthermore, accelerated ageing phenotypes, including secondary cancers, frailty, cognitive impairment and premature mortality, have been reported in cancer survivors across all age groups after treatment (Wang et al., 2021). In addition, the prolonged use of MEK inhibitors, in particular during cancer treatment, can lead to adaptive drug resistance (Kun et al., 2021), with studies reporting that chronic exposure to MEK inhibitors leads to drug resistance in tumour cells associated with upregulation of oncogenic gene expression (Vergani et al., 2020).

Effective strategies to limit these adverse effects will therefore be essential for clinical implementation. Determining the critical time periods during the life course when RAS/MAPK inhibition modulates ageing, and assessing the effect of intermittent dosing, could be one such approach to minimise side effects. Careful titration will also help to provide the geroprotective effects of these drugs without side effects or drug resistance. For example, the effective dose of trametinib that produces geroprotective effects in *Drosophila* is estimated to be lower than those used clinically for cancer treatment (Slack et al., 2015). Additionally, trametinib administration in mice can promote healthy metabolic effects at much lower doses than those used in tumour models (Banks et al., 2015). The effective doses of these compounds as geroprotectors may therefore be much lower than those for cancer treatment. Calibration of therapeutic drug dosage for ageing may not, however, be straightforward. The degree of RAS/MAPK inhibition needed to affect ageing and healthspan is currently unclear. In cancer models, loss of ERK phosphorylation post-treatment is the primary readout for drug potency, and a high degree of ERK inhibition correlates well with treatment efficacy. The same correlation between degree of ERK inhibition and geroprotection has yet to be established in pre-clinical models of ageing.

Identifying the tissues for which RAS/MAPK signalling is limiting for lifespan may also allow for more targeted treatments to reduce side effects. Similarly, further characterisation of the downstream effector targets that mediate the geroprotective response of RAS/MAPK inhibition will provide important evidence of the extent to which inhibiting this pathway could improve health during ageing. Recently, the co-administration of three longevity-promoting compounds, lithium, rapamycin and trametinib, at doses that maximised lifespan extension, extended lifespan in an additive fashion in *Drosophila*. This might be because they produce stronger inhibition of the same targets or because they function at least in part through non-overlapping mechanisms (Castillo-Quan et al., 2019). It would also be interesting to investigate the effect of modulating the function of GAPs to inactivate RAS during ageing, because currently RAS GAPs that promote the RAS-GTP to RAS-GDP exchange have primarily been studied in cancer (Prieto-Dominguez et al., 2019; Gray et al., 2020).

Intriguingly, the pro-longevity effects observed upon genetic inhibition of other cancer promoting pathways suggest that repurposing existing cancer therapeutics as geroprotective agents could extend beyond RAS/MAPK inhibitors. Indeed, recent studies have shown that pharmacological inhibition of AKT using oridonin, a diterpenoid isolated from *Rabdosia rubescens*, prolongs yeast lifespan, extends lifespan and improves multiple parameters of healthspan in mice, including grip strength, cardiac health and kidney function, and inhibits cellular senescence in cultured human fibroblasts (An et al., 2022). A small molecule inhibitor of AKT, GSK690693, also extends *Drosophila* lifespan (Cheng et al., 2022).

Pre-clinical investigations using models of ageing could therefore lead to the development of new drugs or to the repurposing of existing drugs as novel geroprotectors. That either direct or indirect pharmacological inhibition of RAS/MAPK signalling has already been shown to improve healthspan suggests that this approach offers promising new clinical strategies to improve late-life health in humans. Addressing the challenges of clinical translation of these findings, however, is imperative to ensure their safety and success.
